# The Streptococcus pyogenes Signaling Peptide SpoV Regulates Streptolysin O and Enhances Survival in Murine Blood

**DOI:** 10.1128/JB.00586-20

**Published:** 2021-05-07

**Authors:** Andrea L. Herrera, Eduardo A. Callegari, Michael S. Chaussee

**Affiliations:** a Division of Basic Biomedical Sciences, The Sanford School of Medicine of the University of South Dakota, Vermillion, South Dakota, USA; University of Illinois at Chicago

**Keywords:** *Streptococcus pyogenes*, signaling peptide, streptolysin O

## Abstract

Streptococcus pyogenes (group A *Streptococcus* [GAS]) is a human pathogen that causes a wide range of diseases. For successful colonization within a variety of host niches, GAS must sense and respond to environmental changes. Intercellular communication mediated by peptides is one way GAS coordinates gene expression in response to diverse environmental stressors, which enhances bacterial survival and contributes to virulence. Using peptidomics, we identified SpoV (streptococcal peptide controlling virulence) in culture supernatant fluids. SpoV is a secreted peptide encoded near the gene (*slo*) encoding the extracellular cholesterol-dependent cytolysin streptolysin O (SLO). The addition of synthetic SpoV peptide derivatives, but not control peptides, increased *slo* transcript abundance in an M49 isolate but not in an M3 isolate. Deletion of *spoV* decreased *slo* transcript abundance, extracellular SLO protein levels, and SLO-specific hemolytic activity. Complementation of the *spoV* mutant increased *slo* transcript abundance. Lastly, a *spoV* mutant was deficient in the ability to survive in murine blood compared to the parental strain. Moreover, preincubation of the *spoV* mutant with synthetic SpoV peptide derivatives increased GAS survival. Our findings show that *slo* expression is regulated, in part, by the GAS-specific signaling peptide SpoV.

**IMPORTANCE** GAS secretes signaling peptides that can alter gene expression and impact virulence. We used peptidomics to identify a signaling peptide designated SpoV. Further, we showed that SpoV altered the expression of the cholesterol-dependent cytolysin SLO. Peptide signaling plays an important regulatory role during disease progression among several bacterial pathogens, including GAS. The therapeutic potential of manipulating peptide-controlled regulatory networks makes it an attractive option for the development of novel therapeutic strategies that disrupt virulence gene expression.

## Abstract (precis)

GAS secretes signaling peptides that can alter gene expression and impact virulence. We used peptidomics to identify a signaling peptide designated SpoV.

## INTRODUCTION

Streptococcus pyogenes (group A *Streptococcus* [GAS]), is an etiologic agent of pharyngitis and localized skin infections, including impetigo, as well as invasive infections, such as necrotizing fasciitis and streptococcal toxic shock syndrome (1, 2). GAS can also cause serious postinfection sequelae, including acute rheumatic fever and acute poststreptococcal glomerulonephritis (3). The diversity of GAS disease is attributed, in part, to the pathogen’s ability to regulate the expression of a variety of virulence factors, including adherence and invasion proteins, toxins, superantigens, proteases, and immune-modulating proteins (4).

Streptolysin O (SLO) is one of many GAS virulence factors, and it influences pathogenesis in several ways (5–12). First, SLO is a member of the CDC (cholesterol-dependent cytolysin) family of extracellular toxins that lyse eukaryotic cells through the formation of large pores in the membrane (13). Similar to other CDCs, SLO binds to cholesterol-rich areas of the host cell membrane and forms oligomerized complexes in the membrane, creating β-barrel pores up to 30 nm in diameter (13–15). These large pores result in leakage of cytosolic proteins, cell damage, and death (16). Second, SLO facilitates translocation of another GAS toxin, NAD^+^-glycohydrolase (NADase; Nga), into the host cell cytosol in a process termed cytolysin-mediated translocation, in a manner that is not understood (6, 11). Once it is inside the eukaryotic cell, the enzymatic activity of NADase depletes intracellular energy stores (NAD^+^), resulting in cell membrane damage and apoptosis (7, 11, 17). Third, SLO prolongs the intracellular survival of GAS by preventing the maturation of GAS-containing phagosomes by creating pores in the phagosome membrane (9, 18). The pores inhibit lysosomal fusion with the phagosome, which increases intracellular GAS survival (5).Thus, SLO contributes to GAS survival in the human host by multiple mechanisms.

The regulation of GAS virulence factor expression occurs through multiple processes, including those involving peptide-signaling molecules. GAS synthesizes propeptides which are processed to active extracellular signaling peptides during translocation. Extracellular peptides are then imported into the cytosol, where they can interact with transcriptional regulators to alter gene expression in response to changes in the host, including bacterial cell density (19–21). The RRNPP family of quorum-sensing regulators (named for the members: Rap, Rgg, NprR, PlcR, and PrgX) comprises the currently known Gram-positive cytoplasmic quorum-sensing regulators that bind directly to a signaling peptide (22). The RRNPP family is characterized by the presence of a tetratricopeptide repeat (TPR), which consists of a peptide-binding domain (23). Together with a helix-turn-helix (HTH) DNA-binding domain (24), RRNPP regulators activate gene expression following binding to the cognate peptide (19, 22). Other posttranslationally modified peptides produced by GAS include streptococcal invasion locus (*silA*) (25) and salivaricin A (*salA*) (26, 27). Overall, GAS utilizes multiple peptide-signaling systems.

Several GAS signaling peptides influence pathogenesis, including SIP (SpeB-inducing peptide), which controls the production of the secreted virulence factor SpeB. SpeB (streptococcal pyogenic exotoxin B) is a secreted cysteine protease that cleaves or degrades host proteins to contribute to tissue damage and immune evasion (28–33). Under certain conditions, SpeB also cleaves GAS surface proteins, including adhesins, to promote bacterial dissemination (34–38) and contribute further to virulence (39–41). *speB* expression is also influenced by interactions between the N-terminal region of the signaling peptide Vfr (virulence factor-related protein) and the transcriptional regulator Rgg1 (42, 43). Other characterized GAS signaling peptides involved in virulence include Shp2 and Shp3 (short hydrophobic peptides 2 and 3), which induce biofilm production by interacting with the transcriptional regulators Rgg2 and Rgg3, respectively (44). Similar to other pathogens, the ability of GAS to respond effectively to changes in the host environment partially relies on a variety of signaling peptides.

We used peptidomics to identify potential signaling peptides in the culture supernatants of two isolates of GAS. Among the peptides discovered was a peptide encoded by *spyM3_0132.* We showed that the deletion of *spyM3_0132* decreased *slo* transcript abundance, SLO-specific hemolytic activity, and resistance to murine immune effector cells. We named this peptide SpoV, for “streptococcal peptide controlling virulence.”

## RESULTS

### Identification of SpoV in MGAS315 culture supernatant fluids using peptidomics.

**FIG 1:**
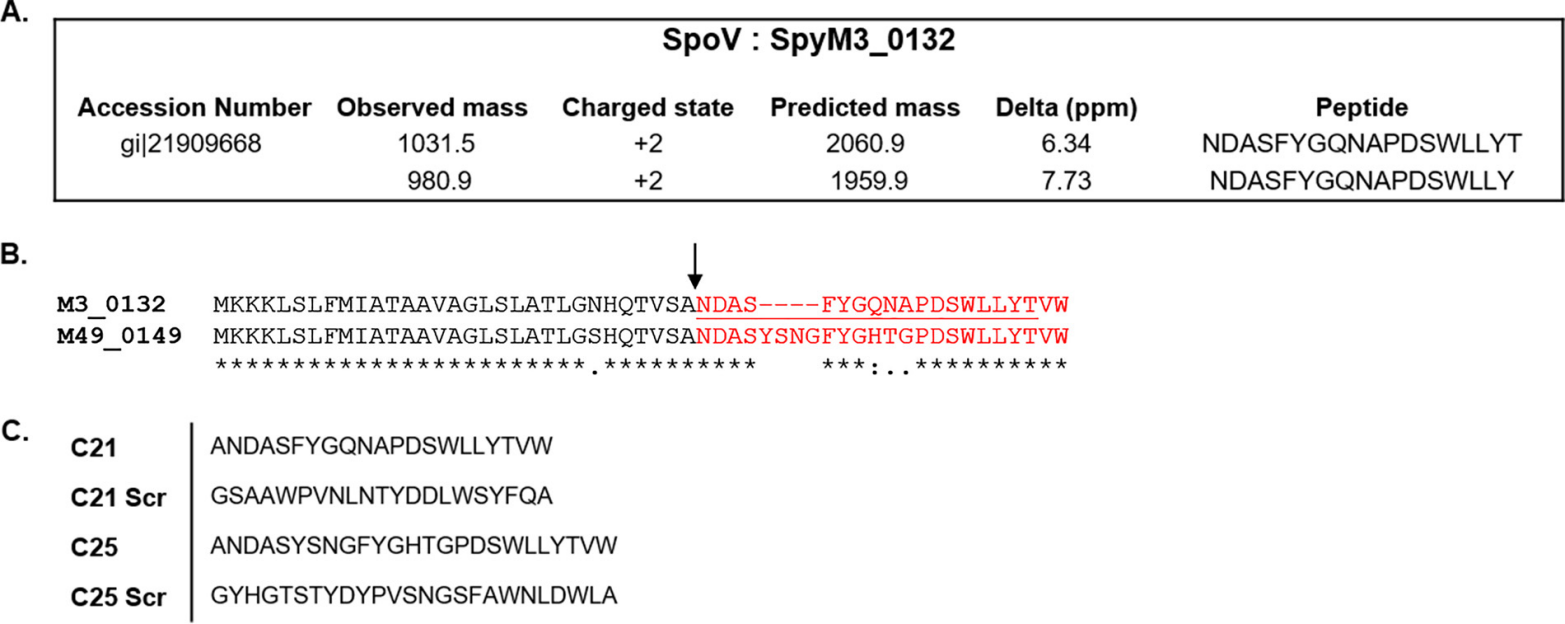
SpoV was identified in MGAS315 culture supernatant fluids using peptidomics. NZ131 and MGAS315 wild-type strains were grown with peptide-free medium overnight. The CSFs were filtered, and peptides were concentrated and analyzed with mass spectrometry. (A) Two peptides corresponding to SpyM3_0132 (designated SpoV) were identified in CSP obtained from MGAS315. (B) Alignment of full-length amino acid sequences of SpoV encoded by strains MGAS315 and NZ131. Asterisks indicate identical residues, colons indicate conserved differences, and periods indicate semiconserved differences. The predicted type II secretory system cleavage site is indicated with an arrow and the corresponding extracellular peptides are shown in red. The peptide identified by mass spectrometry is underlined. (C) Sequence of synthetic peptides containing the 21 C-terminal amino acids of MGAS315 SpoV (C21) or the 25 C-terminal amino acids of NZ131 SpoV (C25) as well as the associated scrambled peptides.

GAS secretes peptides that often have a role in cell-to-cell signaling or quorum sensing. While signaling peptides can be predicted based on nucleic acid sequence, posttranslational processing and a lack of information regarding expression can make it difficult to identify functional signaling peptides. We used peptidomics to identify secreted peptides produced by GAS isolates MGAS315 (serotype M3) and NZ131 (serotype M49). To do so, GAS was grown with peptide-free medium overnight. Peptides in culture supernatant fluids (CSFs) were enriched by using C_18_ reverse-phase cartridges before elution and analysis with mass spectrometry. Among peptides identified in CSF from MGAS315 was a posttranslationally modified peptide encoded by *spyM3_0132*, which we subsequently designated SpoV (Fig. 1; also, see Fig. S1 and S2 in the supplemental material). We did not detect SpoV in CSFs obtained from strain NZ131 (data not shown). The *spoV* open reading frame (ORF) encodes a 51-amino-acid peptide with a type II secretion signal predicted (SignalP 5.0; ExPASy) to result in an extracellular 20-amino-acid peptide in isolate MGAS315. In strain NZ131, a homologous 55-amino-acid peptide is encoded by *spyM49_0149* that is predicted to result in an extracellular 24-amino-acid peptide (Fig. 1B).

A BLASTP search of the National Center for Biotechnology Information (NCBI) database using SpyM3_0132 as a query identified 1,982 similar sequences among GAS isolates. Of 1,982 isolates, 133 (6.6%), including MGAS315, encoded an identical 20-amino-acid extracellular SpoV peptide (NDASFYGQNAPDSWLLYTVW). The other 1,849 (93.4%) isolates, including NZ131, encoded a 24-amino-acid extracellular SpoV peptide. Among the 1,982 isolates, 13 sequence variations exist (Table S1). The most common SpoV sequence (NDASYSNGFYGHTGPDSWLLYTVW) was found among 963 (48.6%) isolates in the NCBI database, including NZ131. The main difference between the extracellular 20- and 24-amino-acid peptides was the presence or absence of the amino acids tyrosine, serine, asparagine, and glycine (YSNG). YSNG was present in the sequences of 1,833 (92.5%) isolates in the database. Orthologues of SpoV were not identified in other bacterial species, including other streptococcal species, suggesting that SpoV has a function specific to GAS.

We synthesized peptides containing the 21 C-terminal amino acids of MGAS315 SpoV (C21) or the 25 C-terminal amino acids of NZ131 SpoV (C25), as well as peptides of the same length and amino acid composition but with a random amino acid sequence (scrambled [C21 Scr and C25 Scr]), for further analysis (Fig. 1C).

### *spoV* transcripts were more abundant in strain MGAS315 than in NZ131.

**FIG 2:**
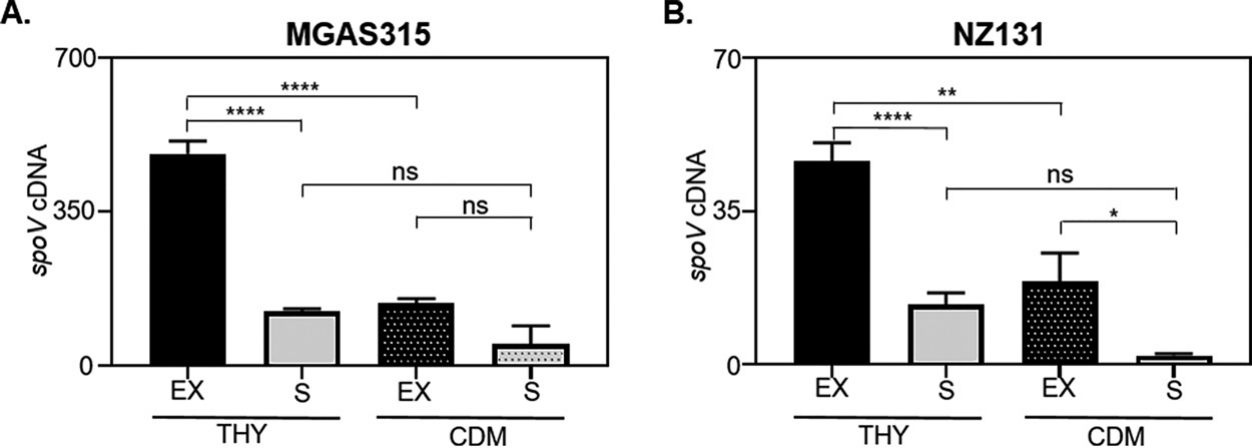
*spoV* transcripts were more abundant in MGAS315 than NZ131. MGAS315 and NZ131 were grown with THY or CDM until the mid-exponential phase (*A*_600_ = 0.4 to 0.7) or stationary phase (*A*_600_ = 0.9 to 1.0). RNA was extracted, and the relative quantity of *spoV* transcripts was determined by qRT-PCR. (A) In MGAS315, *spoV* transcripts were significantly higher in the exponential (EX) phase of growth than in the stationary (S) phase when grown with THY but not when grown with CDM. (B) In NZ131, *spoV* transcripts were significantly higher in the exponential phase than in the stationary phase when grown with either THY or CDM. For both strains, GAS was grown on 4 different occasions, and each RNA sample was analyzed by qRT-PCR in duplicate. Statistical significance was determined by one-way ANOVA with Tukey’s multiple-comparison test. ns, not significant (*P > *0.05); ****, *P < *0.0001; **, *P < *0.01; *, *P < *0.05.

We detected SpoV in CSFs obtained from MGAS315 but not among similarly prepared samples from isolate NZ131. To determine if this was due to differences in expression, we measured the transcript levels of *spoV* in wild-type MGAS315 and NZ131. In addition, we measured expression during the exponential and stationary phases of growth with both peptide-rich medium (Todd-Hewitt yeast [THY]) and peptide-free medium (chemically defined medium [CDM]). During the exponential phase of growth, *spoV* transcripts were 10- and 8-fold higher in MGAS315 than in NZ131 when cultured with THY and with CDM, respectively (*P < *0.0001) (compare Fig. 2A and B). The differences in expression likely explain why the peptide was detected with mass spectrometry among samples obtained from MGAS315 but not NZ131. In THY medium, *spoV* transcripts were more abundant in both strains during the mid-exponential phase than during the stationary phase (*P < *0.0001). Additionally, transcripts of both orthologues were more abundant during growth with peptide-rich medium (THY) than during growth with peptide-free medium (CDM) (*P < *0.0001 for MGAS315; *P < *0.01 for NZ131).

We suspected that the difference in *spoV* expression between MGAS315 and NZ131 was due to differences in the RocA (regulator of *cov*) protein of each strain. MGAS315 has a truncated RocA due to a single nucleotide deletion (45), whereas NZ131 has a full-length RocA. To determine if full-length *rocA* affects *spoV* expression, we transformed wild-type MGAS315 with an Escherichia coli-streptococcal shuttle plasmid (pMRV605) expressing a full-length *rocA* allele. *spoV* transcripts were less abundant in strains expressing a full-length *rocA* allele (MGAS315 WT [pMRV605] and NZ131 wild type [WT]) than in strains expressing a truncated allele [MGAS315 WT and MGAS315 (pAH-VC)] (Fig. S3).

### Synthesized C21 and C25 peptides increased *slo* transcript abundance.

SpoV is encoded proximal to the *slo* gene, which encodes the CDC cytolysin SLO. Based on this observation, we explored the idea that SpoV may influence the expression of *slo*. To do so, we used synthetic SpoV peptides encoded by both strain MGAS315 (C21) and strain NZ131 (C25). C21 and C25 were added separately to MGAS315 or NZ131 cultures grown with peptide-free medium (CDM), and *slo* transcripts were measured by quantitative reverse transcriptase-PCR (qRT-PCR). Scrambled peptides were used as controls. Because the peptides were suspended in dimethyl sulfoxide (DMSO), we also included a control in which the same amount of DMSO was added to the cultures.

**FIG 3:**
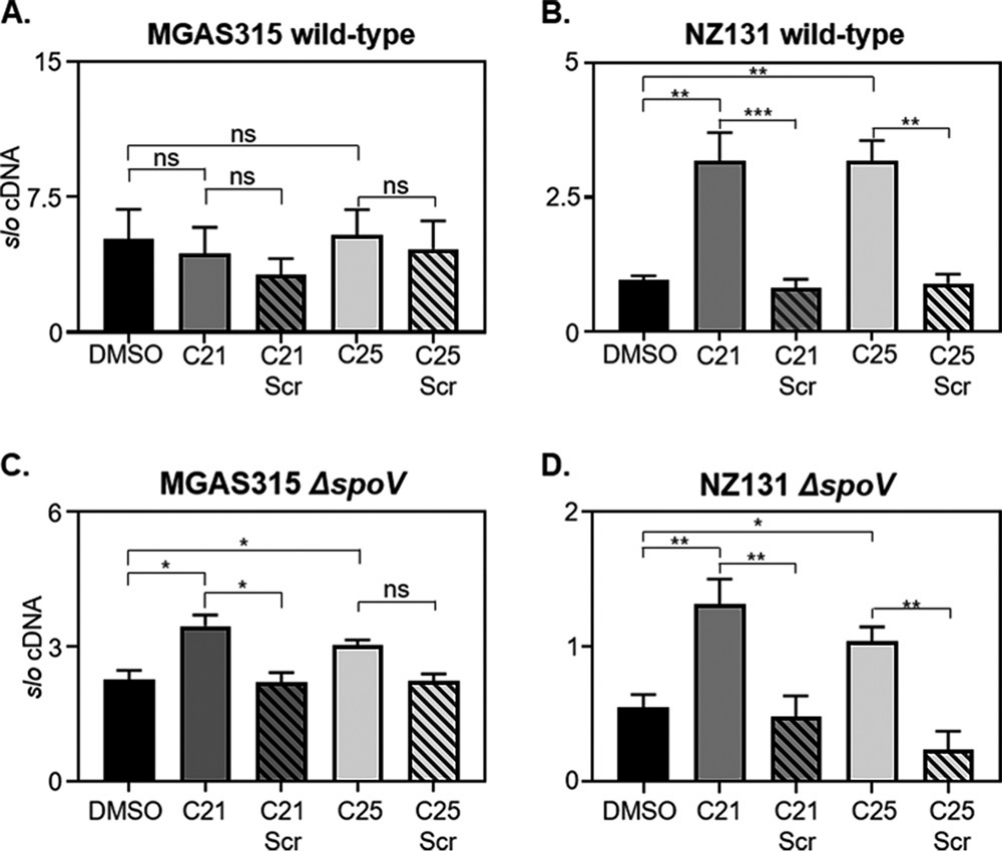
Synthesized C21 and C25 peptides increased *slo* transcript abundance. MGAS315 wild type (A), NZ131 wild type (B), MGAS315 Δ*spoV* (C), and NZ131 Δ*spoV* (D) were grown with peptide-free medium to the mid-exponential phase of growth (*A*_600_, 0.4 to 0.7). Then, a 10 μM concentration of the indicated peptides (C21, C21 Scr, C25, and C25 Scr) or diluent (DMSO) was added to cultures. After 3 h, RNA was extracted, and *slo* transcript abundance was measured by qRT-PCR. RNA was extracted from GAS cultures grown on 3 to 12 different occasions. Each RNA sample was analyzed by qRT-PCR in duplicate. Statistical significance was determined by one-way ANOVA with Tukey’s multiple-comparison test. *P* values were determined by comparison among the indicated strains. ns, not significant (*P > *0.05); ***, *P < *0.001; **, *P < *0.01; *, *P < *0.05.

The addition of neither the C21 nor the C25 peptide to MGAS315 cultures influenced the abundance of *slo* transcripts compared to controls (Fig. 3A). In contrast, the addition of either peptide to NZ131 cultures increased *slo* transcripts compared to controls (Fig. 3B) (*P < *0.01 and *P < *0.001).

To investigate further the role of *spoV* in the regulation of *slo* expression, we created isogenic mutant derivatives of strain MGAS315 and NZ131 in which *spoV* was replaced with a spectinomycin resistance cassette. MGAS315 Δ*spoV* and NZ131 Δ*spoV* were grown with CDM to the mid-exponential phase of growth, and SpoV peptide derivatives (C21, C21 Scr, C25, and C25 Scr) were added. After 3 h, RNA was isolated and *slo* transcripts were measured by qRT-PCR. The addition of the C21 peptide significantly increased the abundance of *slo* transcripts compared to untreated and C21 scrambled controls in both the MGAS315 Δ*spoV* mutant (*P < *0.05) (Fig. 3C) and the NZ131 Δ*spoV* mutant (*P < *0.05) (Fig. 3D). The addition of the C25 peptide significantly increased the abundance of *slo* transcripts compared to untreated and C25 scrambled controls in the NZ131 Δ*spoV* mutant (*P < *0.05) (Fig. 3D). However, the increase in *slo* transcript abundance in MGAS315 Δ*spoV* cultures supplemented with C25 was not statistically significant compared to abundance in MGAS315 Δ*spoV* cultures treated with the scrambled C25 peptide, whereas the increase was significant compared to untreated controls. Overall, the addition of either the C21 or C25 peptide to MGAS315 and NZ131 Δ*spoV* mutants increased the abundance of *slo* transcripts.

We also added the C21 peptide at the time of inoculation and grew MGAS315 Δ*spoV* until mid-exponential phase to determine if (i) adding the C21 peptide earlier or (ii) different concentration of the C21 peptide altered *slo* expression in strain MGAS315 Δ*spoV* (Fig. S4). Adding C21 to MGAS315 Δ*spoV* at the time of inoculation and growing to exponential phase (Fig. S4, bar 1) did not affect *slo* expression compared to adding the C21 peptide to MGAS315 Δ*spoV* during mid-exponential phase of growth (Fig. 3C, bar 2) (*P = *0.7). There was also no difference in *slo* expression among MGAS315 Δ*spoV* samples treated with 10 μM, 50 nM, or 1 nM C21 (Fig. S4). However, adding C21 to MGAS315 Δ*spoV* at all of the concentrations tested increased *slo* expression compared to MGAS315 Δ*spoV* treated with C21 Scr (all concentrations tested) or DMSO. Last, adding 10 nM C21 to MGAS315 Δ*spoV* significantly increased *slo* expression compared to MGAS315 Δ*spoV* treated with 10 μM, 50 nM, or 1 nM C21.

### *spoV* deletion decreased *slo* transcript abundance.

To evaluate further the effect of endogenously produced peptides to the expression of *slo*, we complemented the MGAS315 Δ*spoV* and NZ131 Δ*spoV* strains with the shuttle plasmids (pAH32 or pAH49) which contained the *spoV* ORF of MGAS315 or NZ131, respectively. The ORFs were cloned adjacent to the streptococcal *rofA* promoter (46). A control plasmid (pAH-VC) was also transformed into each GAS isolate to ensure that any differences among the strains were specifically associated with *spoV*.

**FIG 4:**
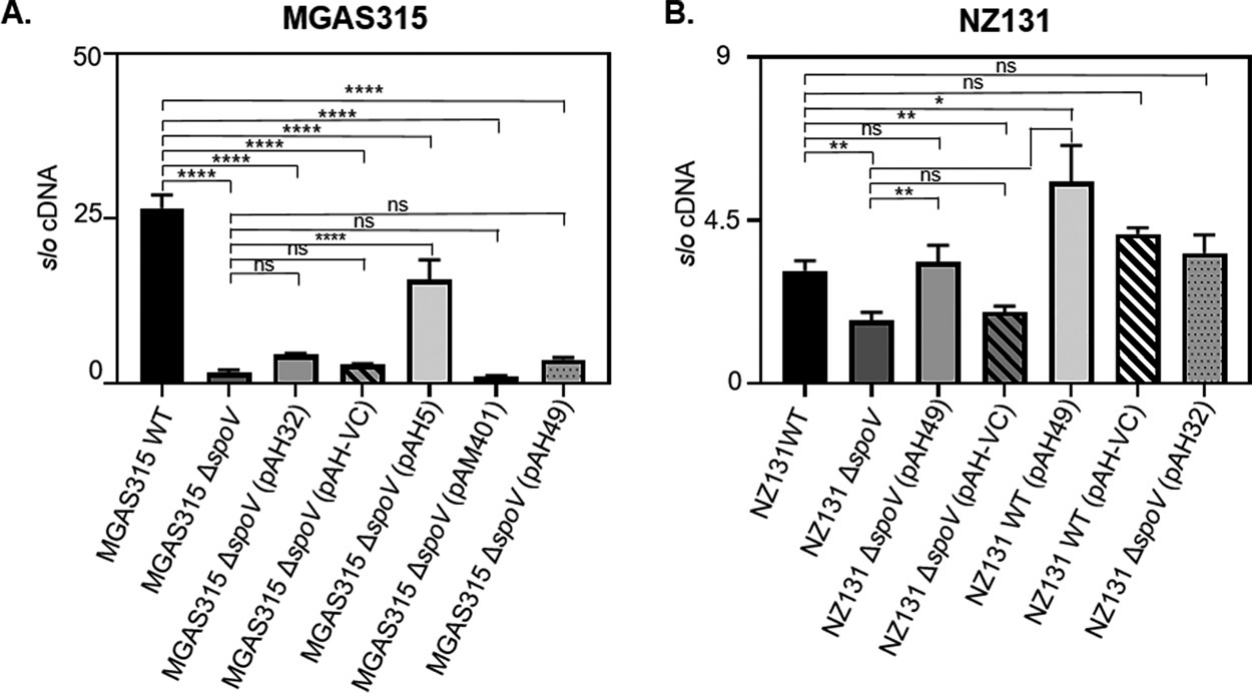
Deletion of *spoV* decreased *slo* transcript abundance. To determine if *spoV* affects *slo* transcript levels, MGAS315 wild type (WT), MGAS315 Δ*spoV*, MGAS315 Δ*spoV* (pAH32), MGAS315 Δ*spoV* (pAH-VC), MGAS315 Δ*spoV* (pAH5), MGAS315 Δ*spoV* (pAM401), MGAS315 Δ*spoV* (pAH49) (A) and NZ131 WT, NZ131 Δ*spoV*, NZ131 Δ*spoV* (pAH49), NZ131 Δ*spoV* (pAH-VC), NZ131 WT (pAH49), NZ131 WT (pAH-VC), and NZ131 Δ*spoV* (pAH32) (B) strains were grown with THY until mid-exponential phase (*A*_600_, 0.4 to 0.7). RNA was extracted from GAS cultures, which were grown on 3 to 13 different occasions. From each RNA sample, *slo* transcripts were measured by qRT-PCR analysis in duplicate. *slo* transcripts were significantly decreased in both MGAS315 Δ*spoV* and NZ131 Δ*spoV* compared to the parental strains. Statistical significance was determined by one-way ANOVA with Tukey’s multiple-comparison test. *P* values were determined by comparison among the indicated strains. ns, not significant (*P > *0.05); ****, *P < *0.0001; **, *P < *0.01; *, *P < *0.05.

Strains were grown with THY, and *slo* transcript levels were measured during the exponential phase of growth. There was a significant 17-fold decrease in *slo* transcripts in MGAS315 Δ*spoV* compared to the parental strain (*P < *0.0001) (Fig. 4A); however, complementation with plasmid pAH32 did not restore *slo* expression to levels observed with the parental strain (*P > *0.05). To determine if the lack of complementation was associated with low expression of *spoV*, we also complemented MGAS315 Δ*spoV* with a pAM401-derived shuttle plasmid, designated pAH5. pAH5 included the *spoV* ORF and 183 bp upstream of the ORF, which was predicted to include an endogenous promoter. Complementation of MGAS315 Δ*spoV* with pAH5 significantly increased *slo* transcript abundance compared to the MGAS315 Δ*spoV* mutant stain (*P < *0.0001).

The abundance of *slo* transcripts in NZ131 Δ*spoV* was significantly lower than that in the parental strain (*P < *0.01) (Fig. 4B). Complementation of NZ131 Δ*spoV* with pAH49 increased *slo* transcript abundance to wild-type levels. Additionally, we transformed the NZ131 wild-type strain with pAH49 to overexpress *spoV* in this isolate. Overexpression of *spoV* (NZ131/pAH49) increased *slo* expression 2-fold (*P < *0.01) compared to expression in the parental strain. Collectively, these results indicated that SpoV increases *slo* expression.

### *spoV* deletion decreased SLO abundance and hemolytic activity.

**FIG 5:**
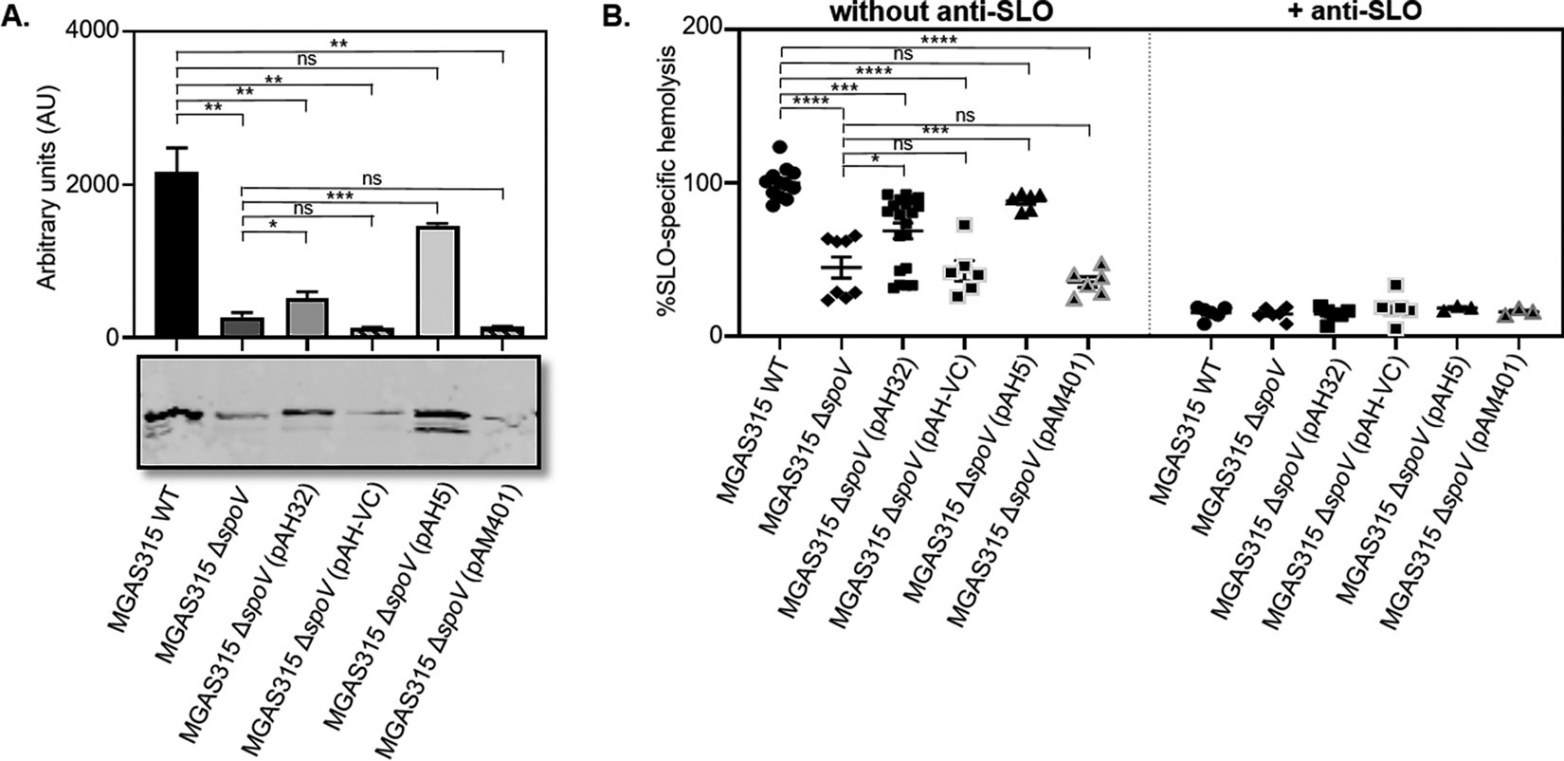
Deletion of *spoV* decreased SLO protein abundance and hemolytic activity. MGAS315 wild-type (WT), MGAS315 Δ*spoV*, MGAS315 Δ*spoV* (pAH32), MGAS315 Δ*spoV* (pAH-VC), MGAS315 Δ*spoV* (pAH5), and MGAS315 Δ*spoV* (pAM401) strains were grown with THY to exponential phase. (A) The amount of SLO present in CSPs was determined by Western blotting using an anti-SLO antibody. Densitometry analysis was used to quantify the results obtained from two independent experiments. A representative image of a blot is shown. (B) SLO-specific hemolysis was determined by measuring the amount of hemoglobin released from erythrocytes. Controls containing 5 % erythrocytes and sterile water, which was considered 100 % hemolysis, were used to determine the percentage of total hemolysis. Results are presented as means and standard errors of the means (SEM). Statistical significance was determined by one-way ANOVA with Tukey’s multiple-comparison test. *P* values were determined by comparison among the indicated strains. ns, not significant (*P > *0.05); ****, *P < *0.0001; ***, *P < *0.001; **, *P < *0.01; *, *P < *0.05.

To determine if the differences in *slo* transcript levels corresponded with the production of SLO, we measured extracellular SLO in MGAS315 and NZ131 culture supernatant proteins (CSPs) by immunoblotting. Consistent with the transcript data, SLO abundance was significantly lower in samples obtained from MGAS315 Δ*spoV* than in those from the parental strain (Fig. 5A). Complementation of MGAS315 Δ*spoV* with either plasmid pAH32 (*P < *0.05) or pAH5 (*P < *0.001) increased SLO abundance, whereas control plasmids did not. In contrast, while overexpression of *spoV* in NZ131 (pAH49) increased SLO abundance compared to wild-type GAS, the increase was not statistically significant (Fig. S5). In addition, statistically significant differences between NZ131 WT and NZ131 Δ*spoV* were not detected (Fig. S5). We also measured hemolytic activity in CSFs obtained from MGAS315 cultures in the presence (Fig. 5B, right) and absence (Fig. 5B, left) of an SLO-neutralizing antibody. Samples from MGAS315 Δ*spoV* had significantly less SLO-specific hemolytic activity than similarly prepared samples from the parental strain. Complementation of MGAS315 Δ*spoV* with either plasmid pAH32 (*P < *0.05) or pAH5 (*P < *0.001) significantly restored hemolytic activity, while complementation with control plasmids did not. There was not a significant reduction in hemolysis with NZ131 Δ*spoV* compared to the wild-type strain; however, a significant increase in hemolysis was detected following overexpression of *spoV* [NZ131 WT (pAH49)] compared to the NZ131 wild type (Fig. S5).

### The deletion of *spoV* decreased GAS survival in murine blood.

**FIG 6:**
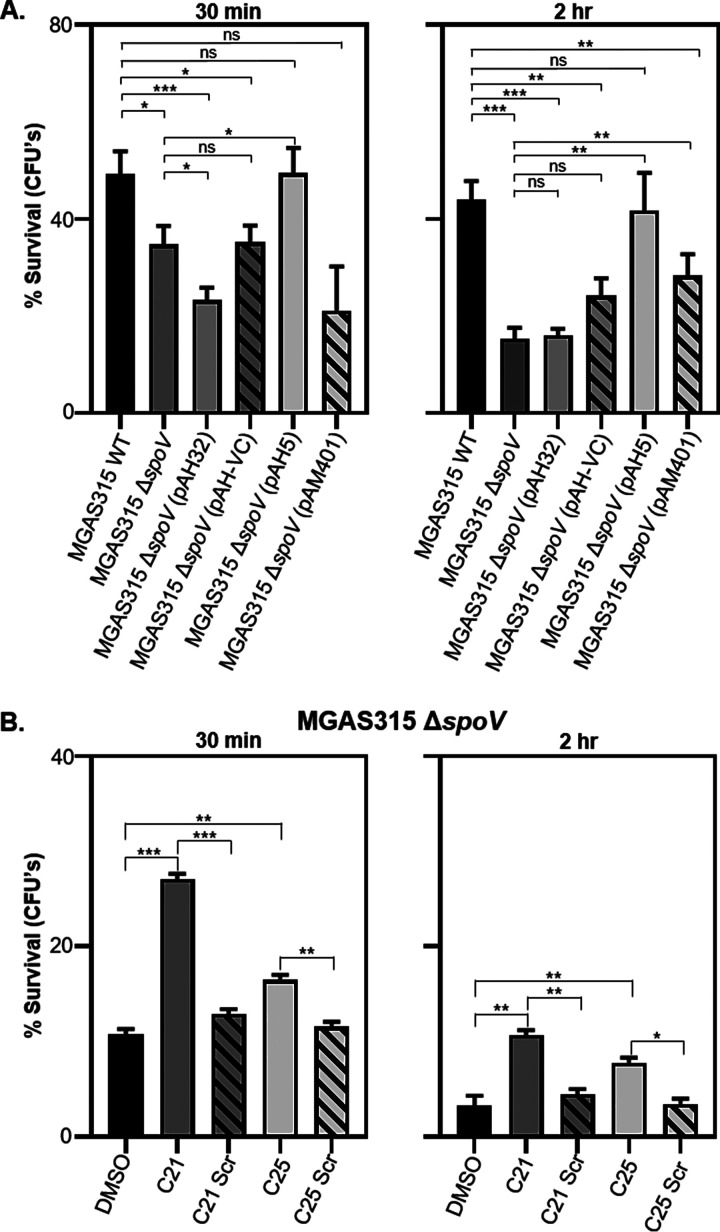
SpoV enhanced GAS survival in whole blood. Approximately 10^4^ CFU of each bacterial strain were mixed with 200 μl of whole murine blood and incubated at 37°C. At specific time points, diluted samples were plated onto agar plates for enumeration. Percent survival is calculated as number of CFU at a given time point divided by the initial number of CFU. (A) Survival of MGAS315 wild type (WT), MGAS315 Δ*spoV*, MGAS315 Δ*spoV* (pAH32), MGAS315 Δ*spoV* (pAH-VC), MGAS315 Δ*spoV* (pAH5), and MGAS315 Δ*spoV* (pAM401) in whole blood. (B) Survival of MGAS315 Δ*spoV* following incubation with 10 μM C21, C21 Scr, C25, and C25 Scr peptides or diluent (DMSO) for 15 min at room temperature. After exposure to the peptides or diluent, the bacteria were mixed with 200 μl whole murine blood and incubated at 37°C. After 30 min or 2 h, diluted samples were plated onto agar plates for enumeration of viable GAS. Statistical significance was determined by one-way ANOVA with Tukey’s multiple-comparison test. *P* values were determined by comparison among the indicated strains. ns, not significant (*P > *0.05); ***, *P < *0.001; **, *P < *0.01; *, *P < *0.05.

To determine if SpoV contributes to the ability of GAS to survive in blood, we suspended MGAS315 strains in whole murine blood and measured GAS viability by dilution plating. The survival of MGAS315 Δ*spoV* was lower than that of wild-type MGAS315 after 30 min (*P < *0.05) and 2 h (*P < *0.001) of exposure to whole blood (Fig. 6A). Complementation of MGAS315 Δ*spoV* with pAH32 did not restore survival in blood after either time point compared to survival of the parental strain. However, complementation with pAH5 significantly increased GAS survival compared to MGAS315 Δ*spoV* after 30 min (*P < *0.05) and 2 h (*P < *0.01).

Additionally, we measured the bacterial survival of MGAS315 Δ*spoV* after incubation with either the C21 or the C25 synthetic peptide (Fig. 6B). After 30 min, 27% (*P < *0.001) and 16% (*P < *0.01) of MGAS315 Δ*spoV* exposed to either C21 or C25, respectively, survived, compared to 11% exposed to the diluent DMSO. After 2 h, 11% (*P < *0.01) and 8% (*P < *0.01) of the initial starting inoculum survived after incubation with C21 and C25, respectively, whereas only 3% of the starting inoculum survived following exposure to DMSO. The results indicated that MGAS315 Δ*spoV* was more resistant to effector cell killing following the addition of C21 or C25 peptide.

## DISCUSSION

GAS is a human-specific pathogen that causes a wide spectrum of diseases. Many isolates secrete peptides that function as signaling molecules and influence the expression of virulence factors. We analyzed secreted peptides produced by isolates MGAS315 and NZ131 using an unbiased peptidomics approach. Among the peptides identified was a putative signaling peptide (SpoV) encoded in the chromosome proximal to the gene encoding the virulence factor SLO. The addition of synthetic SpoV peptide derivatives (C21 or C25) to GAS cultures increased the transcript abundance of *slo* in strain NZ131 but not in MGAS315. Deletion of *spoV* significantly decreased *slo* transcripts in both strains, indicating that SpoV increases *slo* expression. In addition, the abundance of the SLO protein and SLO-specific hemolytic activity were lower in MGAS315 Δ*spoV* mutant than in the parental strain. Last, MGAS315 Δ*spoV* preincubated with synthetic peptide derivatives of SpoV resisted bacterial killing in murine blood more efficiently than strains preincubated with the diluent. The results show that the peptide SpoV contributes to the regulation of *slo* expression in GAS.

SpoV was detected in CSFs obtained from MGAS315 but not NZ131. Consistent with the peptidomics results, the *spoV* transcript levels were less abundant (10-fold) in NZ131 than MGAS315 (Fig. 2). In addition, *slo* expression did not increase in wild-type MGAS315 after the addition of synthetic peptide derivatives, in contrast to the results obtained with strain NZ131 (Fig. 3B). We conclude that because *spoV* is expressed endogenously at a high level in strain MGAS315, compared to NZ131, the addition of more synthetic peptide to MGAS315 cultures did not affect *slo* transcription, since the peptide was already abundant. Additionally, *slo* transcript abundance in NZ131 Δ*spoV* was 1.8-fold lower than in wild-type NZ131. In contrast, there was a 17-fold decrease in *slo* transcript abundance in MGAS315 Δ*spoV* compared to wild-type MGAS315 (Fig. 4). Thus, the effects of *spoV* deletion in NZ131 may be relatively minor, because *spoV* transcript levels are low in this strain. Overall, the results suggest that the quantity of SpoV correlates with *slo* expression.

The CovRS (control of virulence) and RocA (regulator of *cov*) proteins are major regulators of GAS virulence (47). MGAS315 has a mutated *covS* allele (48) and higher expression of virulence genes and virulence (49, 50). Certain GAS serotypes, including M3 serotype isolate MGAS315, encode a truncated RocA variant due to a single nucleotide deletion that results in a frameshift mutation (51). In most other GAS strains, including NZ131, full-length RocA represses the CovRS system, a two-component global regulator of virulence gene expression that influences the transcription of up to 15% of all chromosomal genes (47, 52). Expression of *spoV* was significantly decreased in strains that expressed a full-length *rocA* allele compared to strains that express a truncated *rocA* allele (Fig. S3). *spoV* expression is regulated by RocA, and expression is 637-fold higher in M3 strains with an inactive RocA than in M1 strains with a full-length RocA protein (53). This is consistent with our finding that the serotype M3 strain MGAS315 (with a truncated *rocA*) expresses *spoV* at a higher level than the serotype M49 strain NZ131 (full-length *rocA*) (Fig. 2).

In many two‐component regulatory systems, the response regulator binds to different locations within the promoter region to control target gene expression. CovR binds to the promoter region of many different GAS virulence genes, including the promoters of *has*, *sag*, *speB*, and *ska* (54, 55). CovR also binds to its own promoter and thus regulates its own transcription. There are several copies of the CovR binding site sequence (ATTARA) (56) in the putative promoter region associated with *spoV*, suggesting that *spoV* may be directly controlled by CovR (data not shown). If *spoV* is directly controlled by the CovRS/RocA system, it is likely that other virulence genes or regulatory systems are also affected by the absence or presence of SpoV. For example, the expression of the CovR-regulated cytolysin streptolysin S (SLS) (57) was affected by *spoV* deletion (data not shown), indicating that SpoV likely alters the expression of multiple virulence factors. There were significantly fewer *sagA* transcripts (*P < *0.05) in MGAS315 Δ*spoV*, whereas complementation with pAH32 showed no difference in *sagA* expression compared to the parental strain (*P > *0.05) (data not shown).

GAS can cause invasive diseases, including bacteremia. When GAS is present in the blood, the bacteria may withstand the bactericidal activity of the complement system and phagocytosis by effector cells such as neutrophils and macrophages. Further, host cell damage induces the release of the human antimicrobial peptide LL-37. CovRS senses LL-37, which results in increased expression of GAS virulence factors, including *spoV.* The changes in gene expression increase bacterial resistance to effector cells (58). Consistent with this model, we showed that SpoV increased GAS survival when suspended in whole murine blood (Fig. 6). When SpoV was present (produced either endogenously or after the addition of synthesized SpoV peptides), GAS survived better than did strains lacking SpoV. This indicates that SpoV contributes to the ability of GAS to survive in whole murine blood.

Although SpoV is specific to GAS, the peptide shares some similarity to the peptide PapR of the PlcR/PapR system described for *Bacillus* (59). PlcR (phospholipase C regulator) belongs to the RRNPP family of quorum-sensing regulators. PlcR requires the signaling peptide designated PapR (peptide activating PlcR) to activate target gene transcription (59). PapR is exported from the cytoplasm as an active signaling peptide and then imported to the cytoplasm through the oligopeptide permease (Opp) system (44). Once inside the cytoplasm, PapR binds to the TPR domain of PlcR (44) and promotes binding of PlcR to a defined nucleotide motif termed the PlcR box, which is located upstream of target genes (59, 60). The expression of the CDCs cereolysin O (*clo*) of Bacillus cereus and that of thuringiolysin O (*tlo*) of Bacillus thuringiensis are both regulated by PlcR/PapR (61–63). In contrast, anthrolysin O (*alo*) of Bacillus anthracis contains an upstream PlcR box; however, the PlcR/PapR system does not affect *alo* expression due to a point mutation that truncates PlcR (64).

*papR* is not located in the chromosome near *clo*, *tlo*, or *alo*, whereas *spoV* is downstream of *slo.* Furthermore, we have not identified a CDC that has a signaling peptide encoded proximal to the cytolysin, such as is the case with *spoV* and *slo*. Similar to our results, deletion of *papR* decreases hemolysis in both B. cereus and B. thuringiensis (63, 65) and decreases virulence compared to wild-type B. thuringiensis in an insect larva infection model (59).

The *slo* gene is a part of the *nga-slo* operon, which is transcribed from a promoter upstream of the *nga* gene (11). Regulation of *slo* expression is influenced by different transcriptional regulatory proteins, including CovRS (52), CcpA (66), CodY (67, 68), Rgg1 (69–71), and MsmR (72). Using peptidomics, we identified a GAS signaling peptide named SpoV. SpoV increased the expression of SLO, a critical virulence factor of GAS, and increased the ability of GAS to survive in whole blood (73).

## MATERIALS AND METHODS

### Strain and culture conditions.

Strains used in this study are listed in Table 1. GAS isolates MGAS315 (serotype M3) and NZ131 (serotype M49) were grown at 37°C in a 5% CO_2_ atmosphere with either Todd-Hewitt broth (Becton Dickinson, Sparks, MD) containing 0.2% (wt/vol) yeast extract (THY) or chemically defined medium (CDM). The composition of CDM was previously described, and it lacks peptides (69). Frozen GAS stocks were plated on THY agar and incubated overnight at 37°C in a 5% CO_2_ atmosphere before suspension in liquid medium. A fraction of the suspension was then used to inoculate 10 ml of liquid medium to an *A*_600_ of 0.1, and the cultures were incubated statically at 37°C with 5% CO_2_. Escherichia coli was grown with Luria-Bertani (LB) medium at 37°C with agitation or on LB plates. When appropriate, spectinomycin (Spec; 100 μg/ml for both S. pyogenes and E. coli), kanamycin (Kn; 500 μg/ml for S. pyogenes and 80 μg/ml for E. coli), or chloramphenicol (Cm; 10 μg/ml for S. pyogenes and 50 μg/ml for E. coli) was added to the medium.

**TABLE 1:** Bacterial strains used in this study

Isolate	Genotype	Description
MGAS315	Wild-type (WT)	Serotype M3
	Δ*spoV*	*spoV* mutant (*spoV*::*spec*)
	Δ*spoV* (pAH32)	*spoV-*complemented mutant (*rofA* promoter; MGAS315 *spoV* ORF)
	Δ*spoV* (pAH-VC)	Vector control
	Δ*spoV* (pAH5)	*spoV* complemented mutant (predicted native promoter; MGAS315 *spoV* ORF)
	Δ*spoV* (pAM401)	Vector control
	Δ*spoV* (pAH49)	*spoV-*complemented mutant (*rofA* promoter; NZ131 *spoV* ORF)
	WT (pMRV605)	Full-length *rocA* variant (*rofA* promoter)
	WT (pAH-VC)	Vector control

NZ131	Wild-type (WT)	Serotype M49
	Δ*spoV*	*spoV* mutant (*spoV*::*spec*)
	Δ*spoV* (pAH49)	*spoV-*complemented mutant (*rofA* promoter; NZ131 *spoV* ORF)
	Δ*spoV* (pAH-VC)	Vector control
	Δ*spoV* (pAH32)	*spoV-*complemented mutant (*rofA* promoter; MGAS315 *spoV* ORF)
	WT (pAH49)	*spoV* overexpression (*rofA* promoter)
	WT (pAH-VC)	Vector control

### Construction of deletion mutants.

Deletion mutants of *spoV* were constructed by double-crossover recombination using plasmids that contained a spectinomycin resistance gene (Spec^r^; *aad9*) cloned between DNA sequences identical to those located upstream and downstream of *spoV*.

To delete *spoV* in isolate MGAS315, a plasmid designated pAH3 was synthesized (Integrated DNA Technologies, Coralville, IA) that included DNA sequences 799 bp upstream and 736 bp downstream of the MGAS315 *spoV* ORF.

To delete *spoV* in isolate NZ131, plasmid pAH4 was constructed. First, the plasmid backbone pFW6 (74) (Thermo Fisher) containing two different multiple cloning sites (MCS-I and MCS-II) on either side of *aad9* was synthesized. The 1,033-bp DNA sequence downstream of the NZ131 *spoV* ORF was amplified with PCR and digested with NocI and PstI. The purified amplicon was ligated with the plasmid backbone pFW6, which had also been treated with NocI and PstI (MCS-II). The ligation mixture was transformed into E. coli strain DH5α. Spec^r^ clones were selected, and the resulting plasmid was designated pFW6+DS (pFW6 plus downstream *spoV* fragment). Next, the 740-bp DNA sequence upstream of the *spoV* ORF was amplified with PCR and treated with SfoI and BamHI. The purified amplicon was ligated with pFW6+DS, which had been treated with SfoI and BamHI (MCS-I). The ligation mixture was used to transform E. coli strain DH5α. Spec^r^ clones were selected, and the plasmid was confirmed by DNA sequencing. The resultant plasmid was designated pAH4.

Plasmids pAH3 and pAH4 were linearized, gel purified, and electroporated with MGAS315 or NZ131 wild-type cells (75). Transformants were selected on THY agar plates containing spectinomycin.

### Complementation and overexpression of the *spoV* mutant strains.

*spoV* mutants in both MGAS315 and NZ131 were complemented by expressing the entire *spoV* ORF with a plasmid. To do so, we created plasmids similar to pMNN23, which expresses the transcriptional regulator *rgg1* (*ropB*) using the *rofA* promoter (46). The *rgg1* ORF in pMNN23 was excised with BamHI and PstI to generate the digested plasmid backbone pLZ12 (76). Subsequently, the *spoV* ORF of strain MGAS315 or NZ131 was amplified with PCR using primers that included BamHI and PstI restriction sites, and amplicons were treated with BamHI and PstI. The purified amplicons were then ligated with the purified pLZ12 (76), which had also been treated with BamHI and PstI. The ligation mixture was used to transform E. coli strain DH5α. Kn^r^ clones were selected, and the plasmid constructs were confirmed by DNA sequencing. The resulting plasmids, pAH32 for MGAS315 and pAH49 for NZ131, were used to transform the MGAS315 and NZ131 Δ*spoV* mutant strains, respectively, by electroporation. Additionally, a control plasmid was created using pAH49, by cloning a 100-bp scrambled DNA sequence to replace the *spoV* ORF. The resulting plasmid, pAH-VC (pAH vector control), was used to transform the MGAS315 and NZ131 Δ*spoV* mutant strains by electroporation.

We also complemented the MGAS315 Δ*spoV* mutant by using derivatives of pAM401 with *spoV* expression presumably controlled by the native *spoV* promoter. The *spoV* ORF, as well as the nucleotide sequence beginning 183 bases upstream of the *spoV* start codon was amplified from the MGAS315 genome with PCR and cloned into the pAM401 vector using restriction sites SalI and BamHI which were present within the primers. The recombinant plasmid, pAH5, was then used to transform MGAS315 Δ*spoV* by electroporation, and transformants were selected on agar plates containing chloramphenicol. The MGAS315 Δ*spoV* mutant was also transformed with only pAM401 (pAM401-VC) as a control.

To overexpress *spoV*, pAH49 (described above) was used to transform wild-type NZ131, and Kn^r^ colonies were selected. Wild-type NZ131 was also transformed with pAH-VC (described above) as a control. All strains were confirmed with PCR, DNA sequencing, and qRT-PCR.

### Construction of MGAS315 WT with a full-length *rocA* allele.

The truncated MGAS315 WT *rocA* allele was complemented by expressing the full-length *rocA* ORF with an E. coli-streptococcal shuttle plasmid. To do so, we created a plasmid that expresses full-length *rocA* using the *rofA* promoter (described above). The *rocA* ORF of NZ131 was amplified with PCR using primers that included BamHI and PstI restriction sites, and amplicons were treated with BamHI and PstI. The purified amplicons were then ligated with the purified pLZ12 (76), which had also been treated with BamHI and PstI. The ligation mixture was used to transform E. coli strain DH5α. Kn^r^ clones were selected and the plasmid constructs were confirmed by DNA sequencing. The resulting plasmid, pMRV605, was used to transform MGAS315 WT by electroporation. Additionally, a control plasmid, pAH-VC, was used to transform MGAS315 WT by electroporation.

### Peptide identification.

MGAS315 and NZ131 wild-type strains were grown with CDM medium overnight. After centrifugation (3,220 × *g*, 10 min), the culture supernatant fluids (CSFs) were filtered using a 0.45-μm nylon filter and concentrated with a Centriprep centrifugal filter unit (molecular weight cutoff [MWCO], 3,000 Da) (Millipore) for 15 min (3,220 × *g*, 4°C). Excess salts and other contaminants were removed using a micro-desalting column (Zebra; Thermo Scientific). The peptides were suspended in 100 mM ammonium formate (pH 10) and separated by two-dimensional nano-liquid chromatography (2D-nanoLC) by using a 2D NanoAcquity ultraperformance liquid chromatograph (UPLC) (Waters, Milford, MA). The first dimension was done with an XBridge BEH130 C_18_, 5-μm, 300-μm by 50-mm NanoEase column (Waters, Milford, MA) using solvents A1 (20 mM ammonium formate [pH 10]) and B1 (100% acetonitrile [Fisher Optima; LC-MS grade]). The first-dimension flow rate was 2 μl/min, and 10 different step gradients were performed for 20 min each. The second included trapping and desalting peptides online with a 180-μm by 20-mm, 5-μm Symmetry C_18_ NanoAcquity UPLC trap column (Waters, Milford, MA) at a flow rate of 20 μl/min with 99% A2 (H_2_O, 0.1% formic acid) and 1% B2 (100% acetonitrile, 0.1% formic acid) for 20 min.

After the peptides were desalted and concentrated, they were separated online in the second dimension with a BEH130C18 1.7-μm, 100-μm by 100-mm NanoAcquity UPLC column. The solvent gradient used was as follows: 0 to 2 min, 3% B2 isocratic; 2 to 40 min, 3 to 85% B2 linear at a flow rate of 400 nl/min for 60 min. The eluted ions were analyzed by one full precursor mass spectrometry (MS) scan (400 to 1,500 *m/z*) followed by four MS/MS scans of the most abundant ions detected in the precursor MS scan while operating under a dynamic exclusion or direct data acquisition. Spectra obtained in the positive-ion mode with a nano-electrospray ionization (nano-ESI) Synapt G1 Q-time-of-flight (TOF) HDMS mass spectrometer (Waters, Milford, MA) were deconvoluted and analyzed using MassLynx software 4.1 (Waters, Milford, MA) as well as ProteinLynx Global Server v3.0.3 (PLGS 3.0.3; Waters, Milford, MA), respectively. The peak list (PKL format) was generated to identify +1 or multiple charged precursor ions from the mass spectrometry data file. The instrument was calibrated in MS/MS mode using 100 fmol of (Glu1)-fibrinopeptide B human (Sigma, St. Louis, MO) with a root mean square (RMS) residual of 3.857 e−4 amu or 6.9413 e−1 ppm. Parent mass (MS) and fragment mass (MS/MS) peak ranges were 400 to 1,500 Da and 65 to 1,500 Da, respectively.

### MS/MS database searching.

Mascot server v2.7.0.1 and Mascot Daemon Toolbox v2.5.1 (Matrix Science, UK) in MS/MS ion search mode (local licenses) were applied to conduct peptide matches (peptide masses and sequence tags) and protein searches against the Streptococcus pyogenes genomic database UniProtKB v20191220 (5,161 sequences; 1,459,968 residues). The following parameters were set for the search: no enzyme, no modifications, no missed cleavage; monoisotopic masses were counted; the precursor peptide mass tolerance was set at 10 ppm; fragment mass tolerance was 0.05 Da; and the ion score, or expected cutoff, was set at 5. The MS/MS spectra were searched with MASCOT using a 95% confidence interval (CI) threshold (*P < *0.05), with which a minimum score of 30 was used for peptide identification.

### Synthetic-peptide addition.

Synthetic peptides (>90% purity) were obtained from Biomatik (Fig. 1C) and were suspended in 100% DMSO to prepare a 10 mM stock solution. Stock solutions were aliquoted and stored at −20°C until use. GAS was grown with CDM to the mid-exponential phase of growth, and 10 μl of 10 mM, 1 mM, 50 μM, 10 μM, or 1 μM stock solutions (10 μM, 1 μM, 50 nM, 10 nM, or 1 nM final concentrations) or 10 μl DMSO was added to the cultures. Cultures were incubated at 37°C and 5% CO_2_ for an additional 3 h and then centrifuged and processed for RNA or protein isolation as described below. Scrambled peptides of an identical length and amino acid composition, but differing in the amino acid sequence, were used as controls.

### RNA isolation and qRT-PCR.

To isolate RNA, GAS was grown with 10 ml THY or CDM overnight at 37°C with 5% CO_2_. The cultures were then used to inoculate similar sterile media to an *A*_600_ of 0.1 and incubated at 37°C with 5% CO_2_. Cells were harvested at various time points with centrifugation, suspended in 300 μl RNA later (Thermo Scientific), and frozen at −80°C before RNA isolation. To isolate RNA, cells were disrupted twice for 40 s at speed 6 using a FastPrep homogenizer (Thermo Scientific). A PureLink RNA minikit (Invitrogen) was used to extract and purify RNA samples. RNA samples were treated with turbo-DNase (Ambion) to remove contaminating chromosomal DNA.

Electrophoretic analysis with an Agilent 2100 Bioanalyzer (Agilent Technologies) and the *A*_260_/*A*_280_ ratios were used to assess RNA integrity. Quantitative real-time PCR (qRT-PCR) was done as described previously (77). Briefly, a standard curve was created for each primer set with cycle threshold (*C_T_*) values obtained from the amplification of known quantities of genomic DNA. The standard curves were used to transform *C_T_* values from the qRT-PCR samples to the relative number of transcripts from each sample. All qRT-PCR assays were also performed with RNA templates and no reverse transcriptase to ensure that contaminating DNA was absent.

### CSP isolation.

To isolate CSPs, GAS strains were grown with 40 ml broth at 37°C and 5% CO_2_. The cultures were centrifuged for 15 min at 3,220 × *g* and 4°C to pellet the bacteria. The CSFs were filtered with a 0.2 μM nylon filter, and ice-cold trichloroacetic acid (TCA) and acetone were added to final concentrations of 10% and 5% (vol/vol), respectively. The samples were incubated for 1 h at −20°C before centrifugation for 20 min at 3,220 × *g* and 4°C. The pellets containing CSPs were suspended with 0.5 M Tris containing 5% SDS. Total protein quantitation was determined with the bicinchoninic acid (BCA) protein assay (Thermo Fisher Scientific).

### Western blot analysis of SLO.

CSPs were separated by SDS-PAGE and transferred to nitrocellulose membranes (Amersham; 0.45 μm). For the detection of SLO, a monoclonal anti-streptolysin O antibody (Abcam) was diluted 1:5,000 in 5% nonfat milk and incubated with the membranes for 1 h at room temperature. The membranes were then washed with a solution of phosphate-buffered saline (PBS)–Tween (0.1%) 3 times for 5 min each. The primary antibody was detected using an anti-mouse IgG secondary antibody conjugated with IRDye 700 dye (Li-Cor), which was diluted 1:10,000 in 5% nonfat milk and incubated with the membrane blots for 1 h. The blots were washed and analyzed with densitometry using an Odyssey CLx infrared imaging system with Image Studio imaging software (Li-Cor). Densitometry values (expressed in arbitrary units [AU]) were obtained using the Image Studio imaging software associated with the Li-Cor Odyssey infrared imaging system. To confirm that equal amounts of protein were analyzed, a separate SDS-PAGE gel was stained for total protein using Sypro ruby protein gel stain (Bio-Rad).

### SLO-mediated hemolysis assay.

CSF obtained from mid-exponential-phase cultures was analyzed for SLO hemolytic activity (78, 79). Briefly, sterile CSFs were prepared from 10-ml cultures by centrifugation of the cultures at 3,220 × *g* for 10 min. Supernatant fluids were then filtered through a 0.2-μm nylon filter. Dithiothreitol (DTT) was added to 1 ml of CSF to a final concentration of 2 mM. Then, 50 μl of CSF was added to a 96-well plate with 50 μl of rabbit red blood cells (RBCs), which had been diluted in sterile PBS to a concentration of 5% (vol/vol). Plates were incubated at 37°C for 30 min and centrifuged at 3,220 × *g* for 5 min to pellet intact erythrocytes. Supernatants were transferred to a new 96-well plate, and extracellular hemoglobin from lysed RBCs was measured with *A*_540_. To confirm that hemolysis was due to SLO, a neutralizing polyclonal SLO antibody (Abcam) was incubated with CSFs for 30 min at 37°C before the addition of erythrocytes. In addition, 100% hemolysis was defined with control wells containing 50 μl of RBCs that had previously been diluted to a concentration of 5% RBCs (vol/vol) in sterile water.

### Whole-murine-blood bactericidal assay.

Bacterial strains were grown with THY broth at 37°C to an *A*_600_ of 0.6 to 0.8, centrifuged, suspended in PBS, aliquoted, and stored at −80°C as frozen stocks. The number of viable GAS cells in the stock preparation was enumerated by dilution plating with THY agar plates. Then, the stocks were thawed on ice, and approximately 1 × 10^4^ CFU (diluted in 100 μl PBS) of GAS was added to 200 μl of whole murine blood and incubated at 37°C with rotation. In some experiments, 10 μM C21, C21 Scr, C25, or C25 Scr peptides or diluent (DMSO) was added directly to GAS strains, which had been thawed and diluted to 1 × 10^4^ CFU for 15 min at room temperature. After exposure to the peptides or diluent, the bacteria were mixed with 200 μl whole murine blood and incubated at 37°C. Diluted blood samples were plated onto agar plates at selected time points to enumerate the surviving bacteria. The survival rate was calculated as (CFU at a given time point/CFU at the start) × 100.

### Statistics.

All quantification and statistical analysis of data were done with GraphPad Prism 8 software. The statistical analyses included a one-way analysis of variance (ANOVA) with a Tukey multiple-comparison *post hoc* test. Values were accepted as significant if the *P* value was less than 0.05.

## Supplementary Material

Supplemental file 1
