# Supplementary material for: The Streptococcus pyogenes Signaling Peptide SpoV Regulates Streptolysin O and Enhances Survival in Murine Blood
Source: J Bacteriol. 2021 May 7;203(11):e00586-20. doi: 10.1128/JB.00586-20 (PMC8117530; doi:10.1128/JB.00586-20)
Supplement: Supplemental file 1 [file JB.00586-20-s0001.pdf]

TABLE S1

# of GAS isolates per variation	Extracellular SpoV sequence (amino acid length)		
133	**NDAS----FYGQNAPDSWLLYTVW**	**(20)**	***MGAS315***
963	....YSNG...HTG..........	(24)	NZ131
569	....YSNG...H.G..........	(24)	
155	..T.YSNG...HTG..........	(24)	
101	....YSNG........N.......	(24)	
17	....YSNG...H.G.E........	(24)	
14	...SYSN....H.G..........	(24)	
11	...NYSNG...H.G.E........	(24)	
5	....YSNG...HT...........	(24)	
5	....YSNG....TG..........	(24)	
5	....YSNG...PTSS...V.....	(24)	
2	....YSNV...H.G..........	(24)	
1	..T.YSNG...HTG.......I..	(24)	
1	.E..YSNG...HTG..........	(24)	
Total = 1,982			

TABLE S1. Amino acid sequence variation of the predicated extracellular portion of SpoV. Residues highlighted in red are variations of the SpyM3_0132 sequence. The number of GAS isolates with a particular SpoV sequence is listed in the left column.

TABLE S2

Primer name	Sequence (5’----3’)	Purpose
qPCR_slo_F	GTAGTCACCAAGCGAACCC	qRT-PCR of slo
qPCR_slo_R	GGCAGGAAGCGATTACCAC	qRT-PCR of slo
qPCR_spoV_F	ACCACCAAACTGTTTCAGCA	qRT-PCR of spoV
qPCR_spoV_R	TAACCAGCTGTCAGGAGCAT	qRT-PCR of spoV
qPCR_sagA_F	CAAGTTGCTCCTGGAGGCT	qRT-PCR of sagA
qPCR_sagA_R	CTTCCGCTACCACCTTGAGA	qRT-PCR of sagA
Upstream 0149_SfoI_F	GGCGCGGCGCCAAAGAGTTTGTGAAAGAGTCTCTC	pAH4
Upstream 0149_BamHI_R	CGATGGATCCGCCTAATACCGTAACATAGAAGCTATCTTC	pAH4
Downstream 0149 _NcoI_F	GGCGCCCATGGAGATAACTCCTTTAATTTGTAGTATGACTATA	pAH4
Downstream 0149 _Pst_R	CGATCTGCAGAACCGTATAGCCATGTAATAAAGTAGTGTTTTC	pAH4
spoV_ORF_BamHI_F	GGCGCGGATCCATGAAAAAGAAATTATCTTTATTTATGATCGCAAC	pAH32 & pAH49
spoV_ORF_PstI_R	CGATCTGCAGTTACCATACAGTGTAAAGTAACCAG	pAH32 & pAH49
spoV_ORF +183bp_SalI_F	CGATGTCGACACTAGTCACAGCATAGTCTACC	pAH5
spoV_ORF +183bp_BamHI_R	CGATGGATCCTTACCATACAGTGTAAAGTAACCAG	pAH5
rocA_BamHI_F	TAAGCAGGATCCATGTTAGAAGATTTTCTTCAATTTTT	pMRV605
rocA_PstI_R	TAAGCACTGCAGTCAGTCAGGCTTAGCTATT	pMRV605

TABLE S2: primers used for cloning and qRT-PCR

Some primers were synthesized to incorporate restriction endonuclease cleavage sites (underlined and bold). F, forward; R, reverse.

FIG. S1

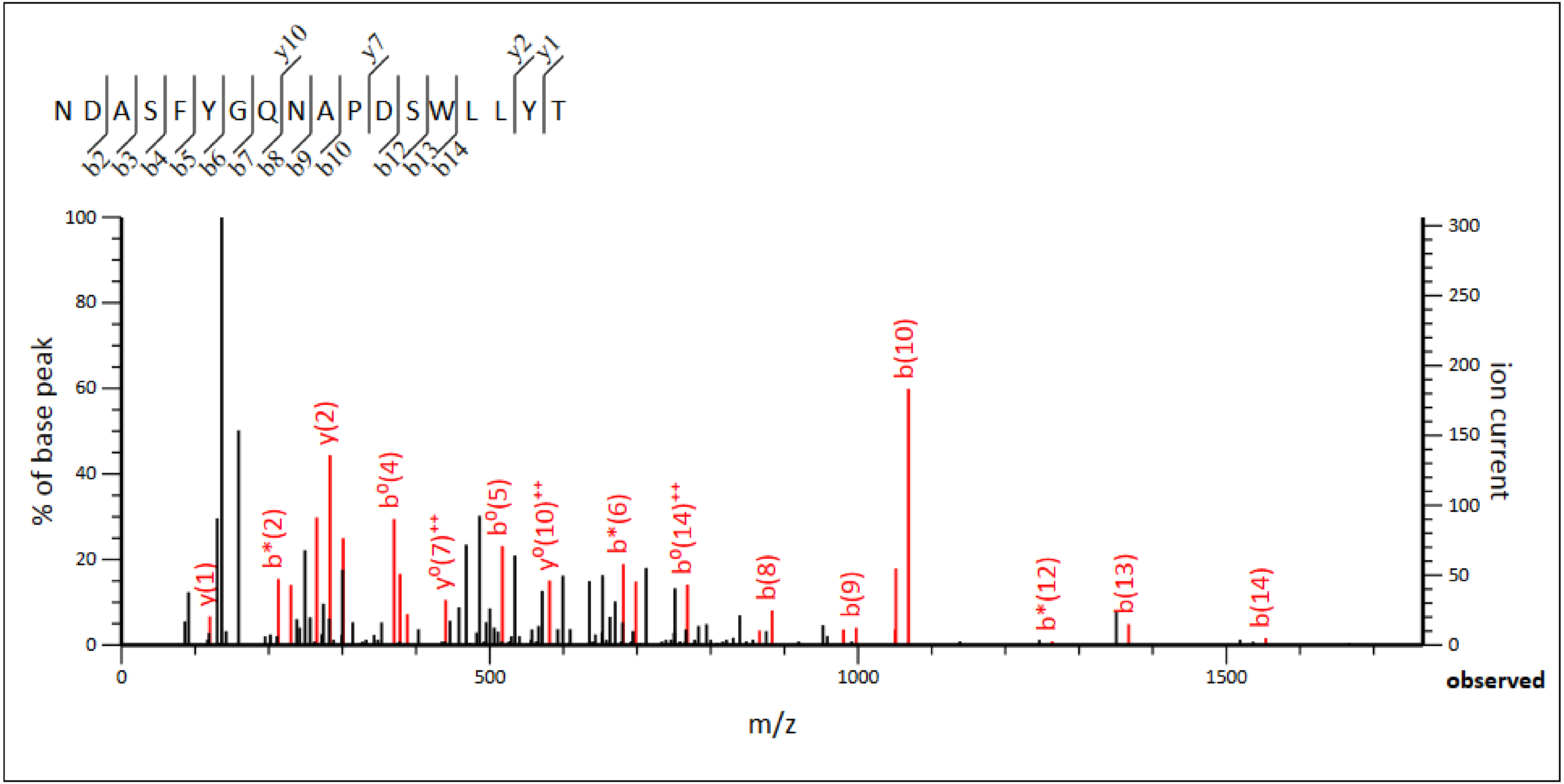

FIG. S1: MS/MS fragmentation map of NDASFYGQNAPDSWLLYT (2060.92465 Da) from m/z = 1031.46960, 2+ (Mascot ion mode search) precursor ion.

FIG. S2

#	b	b^{++}	b^{*}	b^{*++}	b^{0}	b^{0++}	Seq.	y	y^{++}	y^{*}	y^{*++}	y^{0}	y^{0++}	#
1	115.0502	58.0287	98.0237	49.5155			N							18
2	***230.0771***	115.5422	213.0506	107.0289	212.0666	106.5369	D	1947.8759	974.4416	1930.8494	965.9283	1929.8654	965.4363	17
3	***301.1143***	151.0608	284.0877	142.5475	283.1037	142.0555	A	1832.8490	916.9281	1815.8224	908.4149	1814.8384	907.9229	16
4	***388.1463***	194.5768	371.1197	186.0635	370.1357	185.5715	S	1761.8119	881.4096	1744.7853	872.8963	1743.8013	872.4043	15
5	535.2147	268.1110	518.1882	259.5977	517.2041	259.1057	F	1674.7799	837.8936	1657.7533	829.3803	1656.7693	828.8883	14
6	***698.2780***	349.6427	681.2515	341.1294	680.2675	340.6374	Y	1527.7114	764.3594	1510.6849	755.8461	1509.7009	755.3541	13
7	755.2995	**378.1534**	738.2729	369.6401	737.2889	369.1481	G	1364.6481	682.8277	1347.6216	674.3144	1346.6375	673.8224	12
8	***883.3581***	442.1827	866.3315	433.6694	865.3475	433.1774	Q	1307.6266	654.3170	1290.6001	645.8037	1289.6161	645.3117	11
9	***997.4010***	499.2041	980.3745	490.6909	979.3904	490.1989	N	1179.5681	590.2877	1162.5415	581.7744	1161.5575	581.2824	10
10	***1068.4381***	534.7227	1051.4116	526.2094	1050.4275	525.7174	A	1065.5251	533.2662			1047.5146	524.2609	9
11	1165.4909	583.2491	1148.4643	574.7358	1147.4803	574.2438	P	994.4880	497.7477			976.4775	488.7424	8
12	1280.5178	640.7625	1263.4913	632.2493	1262.5073	631.7573	D	897.4353	449.2213			879.4247	440.2160	7
13	***1367.5498***	684.2786	1350.5233	675.7653	1349.5393	675.2733	S	782.4083	391.7078			764.3978	382.7025	6
14	***1553.6292***	777.3182	1536.6026	768.8049	1535.6186	768.3129	W	695.3763	348.1918			677.3657	339.1865	5
15	1666.7132	833.8603	1649.6867	825.3470	1648.7027	824.8550	L	509.2970	255.1521			491.2864	246.1468	4
16	1779.7973	890.4023	1762.7707	881.8890	1761.7867	881.3970	L	396.2129	198.6101			378.2023	189.6048	3
17	1942.8606	971.9339	1925.8341	963.4207	1924.8501	962.9287	Y	**283.1288**	142.0681			265.1183	133.0628	2
18							T	**120.0655**	60.5364			102.0550	51.5311	1

FIG. S2. Monoisotopic mass of neutral peptide Mr (calc); 2060.9116. Fragment ions mass (b and y series) obtained with MS/MS and corresponding to NDASFYGQNAPDSWLLYT (numbers in red represent the significant fragments with a score > 30; *p* <0.05, from Mascot MS/MS ion search mode).

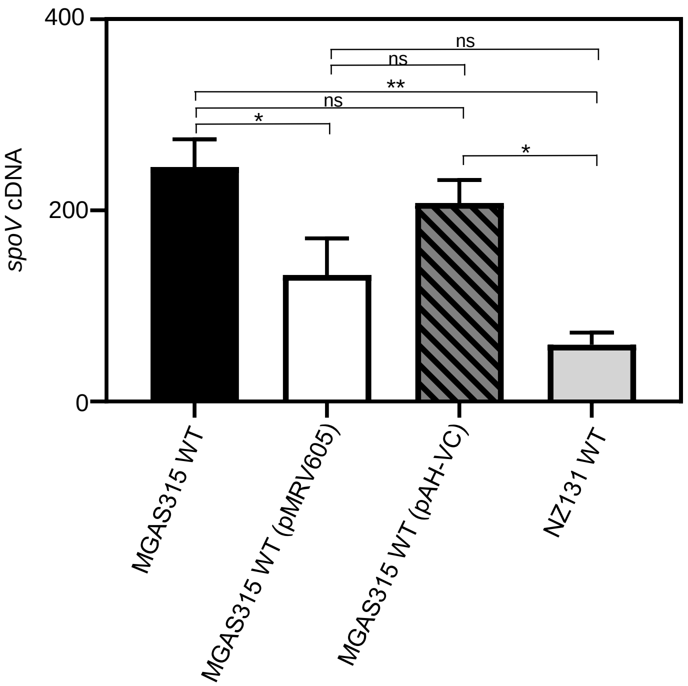

FIG. S3. *spoV* transcripts were less abundant in strains expressing a full-length *rocA* allele. MGAS315 wild-type, MGAS315 WT (pMRV605), MGAS315 (pAH-VC), and NZ131 WT were grown with THY until the mid - exponential (A_{600} = 0.4 - 0.7) phase of growth. RNA was extracted and the relative quantity of *spoV* transcripts was determined with qRT-PCR. In MGAS315 WT (pMRV605) *spoV* transcripts were significantly lower compared to the parental strain. For all strains, GAS was grown on 4 different occasions and each RNA sample was analyzed with qRT-PCR in duplicate. Statistical significance was determined by using the One-way ANOVA test with Tukey's multiple comparison test. (*p* values; ns, not significant [$p > 0.05$]; **, $p < 0.01$; * $p < 0.05$).

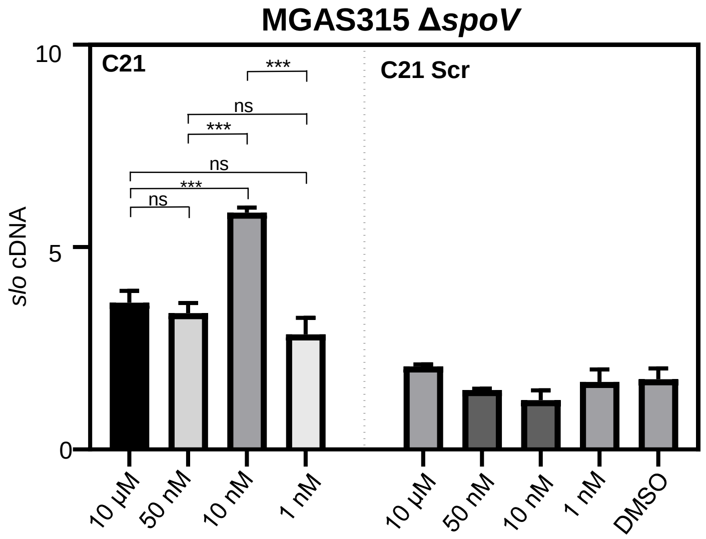

FIG. S4. Synthesized C21 peptide increased *slo* transcript abundance. MGAS315 Δ*spoV* was grown with peptide-free media. At the time of inoculation 10 µM, 50 nM, 10 nM, or 1nM of the indicated peptides (C21, C21 Scr) or diluent (DMSO) were added to cultures. After strains reached early exponential phase of growth, RNA was extracted and *slo* transcript abundance was measured with qRT-PCR. Each RNA sample was analyzed with qRT-PCR in duplicate. Statistical significance was determined by using the One-way ANOVA test with Tukey's multiple comparison test. *p* values were determined by comparison amongst the indicated strains (*p* values; ns, not significant [$p > 0.05$]; ***, $p < 0.001$; **, $p < 0.01$; * $p < 0.05$).

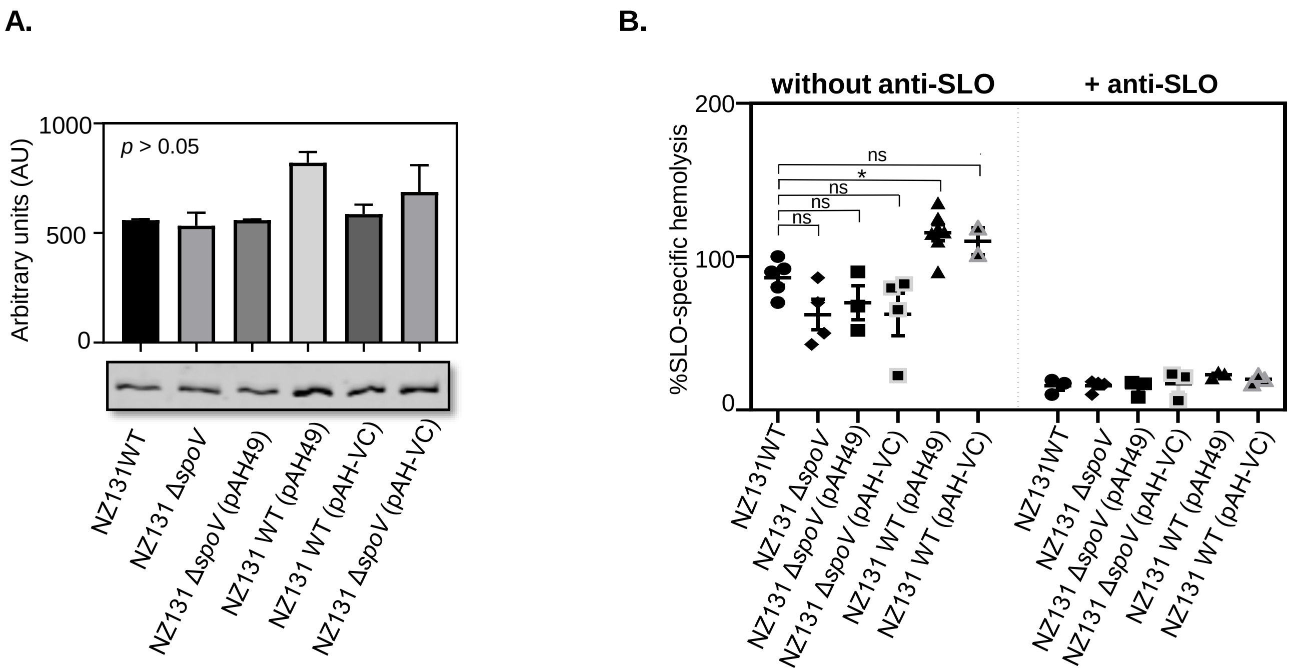

Fig. S5. Overexpression of *spoV* increased SLO hemolytic activity. NZ131 wild-type (WT), NZ131 Δ*spoV*, NZ131 Δ*spoV* (pAH49), NZ131 WT (pAH49), NZ131 WT (pAH-VC), and NZ131 Δ*spoV* (pAH-VC) strains were grown with THY to the exponential phase of grown. **A.** The amount of SLO present in CSPs was determined by western blotting using an anti-SLO antibody. Densitometry analysis was used to quantify the results obtained from three independent experiments. A representative image of a blot is shown. **B.** SLO-specific hemolysis was determined by measuring the amount of hemoglobin released from erythrocytes. Samples were incubated with or without anit-SLO to inhibit SLO hemolytic activity. Controls containing 5% erythrocytes and sterile water, which was considered as 100% hemolysis, were used to determine the percentage of total hemolysis. Results are presented as the mean of ± SEM. Statistical significance was determined by using the One-way ANOVA test with Tukey's multiple comparison test. p values were determined by comparison amongst the indicated strains (p values; ns, not significant [$p > 0.05$]; * $p < 0.05$).
